# Influence of Musculoskeletal System Dysfunction Degree on Psychophysiological Indicators of Paralympic Athletes

**DOI:** 10.3390/sports7030055

**Published:** 2019-02-26

**Authors:** Zhanneta Kozina, Maryna Lytovchenko, Danylo Safronov, Yurii Boichuk, Olena Chaika, Tatiana Shepelenko, Anton Polianskyi, Victor Protsevskiy, Lyudmila Peretyaha, Maya Konnova

**Affiliations:** 1 Department of Olympic and professional sport and sport games, H.S. Skovoroda Kharkiv National Pedagogical University, 61002 Kharkiv, Ukraine; marylytovchenko@gmail.com; 2 Department of Surgical Diseases and Topographic Anatomy of Kharkov National University, 61002 Kharkiv, Ukraine; safronovdanil70@gmail.com; 3 Department of Human Health and correctional education, H.S. Skovoroda Kharkiv National Pedagogical University, 61002 Kharkiv, Ukraine; yurij.boychuk@gmail.com; 4 Department of Physical Education, Kharkov National Air Force University, 61002 Kharkiv, Ukraine; helen04011981@ukr.net; 5 Department of Physical Education and Sport, Ukrainian State University of Railway Transport, 61002 Kharkiv, Ukraine; shepelenko_tatyana@ukr.net; 6 Department of Criminal Law Disciplines, H.S. Skovoroda Kharkiv National Pedagogical University, 61002 Kharkiv, Ukraine; anton_polianskyi@mail.ru (A.P.); protsevskyi0905@gmail.com (V.P.); 7 Department of Bringring Up and Technology of Pre-School Education, H.S. Skovoroda Kharkiv National Pedagogical University, 61002 Kharkiv, Ukraine; leperetyagavvmakarov@gmail.com; 8 Department of Management and Administration, Vinnytsia Academy of Continuing Education, 21001 Vinnytsia, Ukraine; mkonnova@gmail.com

**Keywords:** table tennis, athletes, Paralympians, musculoskeletal, psychophysiological indicators

## Abstract

The purpose of the work was to identify the influence of functional class and degree of damage to extremities on psychophysiological indicators of Paralympians. The study involved 33 elite athletes with musculoskeletal system disorders of the 6 (*n* = 15) and 10 (*n* = 18) functional classes in table tennis, aged 21–25 years old. Parameters characteristic for determining the psychophysiological state and typological characteristics of the nervous system were analyzed with the help of computer programs for psychophysiological testing. We determined the latent time of simple and complex reactions in different testing modes. Dispersion analysis was also used. We applied single-factor multidimensional dispersion analysis: one-way analysis of variance and General Linear Model, Multivariate. The indicators of psychophysiological testing were applied as dependent variables. The values of the functional class of athletes were used as the independent variable. To study the influence of damage degree of the upper or lower extremities on psychophysiological indicators, the extremities damage degree was applied as an independent variable. The time in the Paralympic 6 functional class to reach the minimum signal exposure in feedback mode was significantly longer compared with the 10 Paralympic functional class (*p* < 0.05). Comparing psychophysiological indicators when Paralympians are divided into groups more differentiated than functional classes (that is, according to the nature of the disease or the degree of limb lesions), significant differences were found in all psychophysiological indicators between the athletes of different groups. The greatest impact on psychophysiological indicators was a lesion of the lower extremities. The training of Paralympians in table tennis should consider the reaction rate indicators. In addition, when improving the functional classification of Paralympians in table tennis, a more differentiated approach should be taken when considering their capabilities, including psychophysiological indicators. During training and functional classification of Paralympic athletes in table tennis, it is important to consider their functional class as well as the degree of damage to upper and lower extremities and the level of psychophysiological functioning.

## 1. Introduction

Paralympic sport is becoming an increasingly significant aspect in society [1,2]. The level of sportsmanship of the Paralympic Games participants is constantly increasing, as is the level of Paralympic athletic performance athletes [3,4,5]. Any disability poses problems for adapting to life in a new capacity [6] given the need to master vital and professionally necessary motor skills and habits, and the need to develop and improve special physical and volitional qualities and abilities. The social, spiritual, and moral importance of sports competitions with the participation of athletes with disabilities is obvious [7,8]. 

The training of Paralympians has specific characteristics. These features are specific to each kind of sport [9]. Each athlete with musculoskeletal system dysfunction also has their own characteristics in terms of various motions and adaptation to society. Congenital typological features of the nervous system are added to the athletes’ individual characteristics of motor abilities [10]. These indicators determine the individual characteristics of athletes [11]. The identification of these indicators is related to the field of psychophysiological research. Psychophysiology is the branch of psychology concerned with the physiological bases of psychological processes [12]. Mental chronometry is studied using measurements of reaction time (RT), which is the elapsed time between the presentation of a sensory stimulus and the subsequent behavioral response and is an aspect involved in experimental psychophysiology [13]. Mental chronometry is the use of response time in perceptual-motor tasks to infer the content, duration, and temporal sequencing of cognitive operations. The results of mental chronometry were used as indicators of psychophysiological functions.

Muscular activity is controlled by the central nervous system [13]. Therefore, there is an interconnection between the working of the nervous system and that of the musculoskeletal system [14]. Disorders in the working of the musculoskeletal system affect the working of the central nervous system. This may be important for activities that require a high level of nervous system reactivity [14,15], such as sports games (basketball, football, volleyball, handball, tennis, rugby, and others) and martial arts [16,17,18]. One of the sports that requires a high level of the nervous system reactivity is table tennis, including high reaction rate and high mobility of the nervous system. This is important for both Olympians and Paralympians. Therefore, an aspect of Paralympian training involves studying the characteristics of the reaction rate and the nervous system mobility in athletes with different levels of musculoskeletal system damage [19,20,21]. 

Many studies have been devoted to the central nervous system of persons with disabilities [22,23,24]. Arnold et al. [25] showed that there are differences and similarities in stress factors in Olympians and athletes with disabilities. These studies demonstrated the effect of musculoskeletal system disorders on the psychology of perception of the surrounding world. These effects were confirmed by many other studies [26,27,28]. The study of psychophysiological adaptation mechanisms is necessary to strengthen individuals and prevent athletic injuries [29]. 

Determining the features of psychophysiological functions (reaction rate and mobility of nervous processes) is important for the implementation of an individual training program for table tennis Paralympians [20]. The competitive activity of Paralympic athletes in table tennis is regulated by a classification through which athletes are divided into functional classes depending on the musculoskeletal system damage degree [30]. Functional classes unite athletes not by the nature of the disease, but by the level of function required for playing table tennis [30]. Therefore, within each functional class, there are large differences between athletes in the nature of the disease, the volume of affected muscles, and the functioning of the nervous system. These differences considerably influence the features of the training process and competitive activities. For the rational arrangement of the table tennis training process for Paralympians, their functional class and the nature of the disease and the volume of affected muscles need to be considered.

In the literature, data have been reported concerning the interaction of muscles and the nervous system [31,32]. An important challenge is to determine whether Paralympians with different damage degrees of the musculoskeletal system differ in psychophysiological indicators. To do this, elite Paralympians in various functional classes can be compared according to the classification in the Paralympic table tennis. However, since Paralympians differ not only in functional class but also in the nature of the musculoskeletal system disorder and the volume of affected muscles, athletes should also be compared according to these characteristics.

The degree of musculoskeletal system disorder is determined by different scales [33,34]. The muscle strength [35], balance [36], general functionality [37], and risk of falls [38,39] are also considered. As such, Paralympians that are representative not only of functional classes but also of different musculoskeletal system damage should be compared. Identifying the psychophysiological functions features of Paralympians with different levels and features of the musculoskeletal system damage, specializing in table tennis, will allow the creation of a more precise and individualized training process. These data may be also useful for improving the functional classifications of Paralympians.

One of the main psychophysiological indicators is the reaction rate in various testing modes and typological features of the nervous system. Based on the analyzed literature, our hypothesis is the following: psychophysiological indicators are different in Paralympians with different levels of musculoskeletal system damage. The purpose of the work was to identify the influence of the functional class and the degree of damage to extremities on psychophysiological indicators of Paralympians.

## 2. Materials and Methods

### 2.1. Participants

The study involved 33 elite male athletes with the musculoskeletal system disorders of the 6 (*n* = 15) and 10th (*n* = 18) functional classes in table tennis, aged 21–25 years. The study was carried out in accordance with the principles of the Helsinki Declaration and approved by the Ethics Committee of the H.S. Skovoroda Kharkiv National Pedagogical University, Kharkiv, Ukraine. 

### 2.2. Characteristics of Athletes 

Minimum impairment for athletes was competed in a standing position with cerebral palsy, amputations, and other lesions of the musculoskeletal system [30] (Appendix A).

### 2.3. Division of Athletes According to Upper and Lower Limb Activity Degree 

In addition to the functional classification, we also divided the athletes according to the upper and lower limb activity degree. This division was carried out for the athletes with a predominant upper limb impairment and a predominant lower limb impairment because these impairments are associated with different parts of the central nervous system. 

#### Instruments in the Study

We developed a special scale is based on existing scales for assessing musculoskeletal system disorders and are applied in rehabilitation.

The scale used for muscular strength assessing was the Scale for Assessing Muscle Strength [35]. Patterns of weakness can help localize a lesion to a particular cortical or white matter region, spinal cord level, nerve root, peripheral nerve, or muscle. This scale involves testing the strength of each muscle group and recording it in a systematic fashion. The testing of each muscle group should be immediately paired with testing of its contralateral counterpart to enhance detection of any asymmetries. Muscle strength is often rated on a scale of 0.5 to 5.5.

The scale we used for balance assessment was the Berg Balance Scale (BBS) [36]. The BBS is a qualitative measure that assesses balance via performing functional activities, such as reaching, bending, transferring, and standing, that incorporates most components of postural control: sitting and transferring safely between chairs; standing with feet apart, feet together, in single-leg stance, and feet in the tandem; Romberg position with eyes open or closed; and reaching and stooping down to pick something off the floor. Each item is scored on a 5-point scale, ranging from 0 to 4, each grade with well-established criteria. Zero indicates the lowest level of function and 4, the highest level of function. The total score ranges from 0 to 56. The BBS is reliable (both inter- and intra-tester) and has concurrent and construct validity (validity coefficient = 0.78; validity coefficient = 0.74, respectively) [36].

The scale we used for functional capacity determination was the Functional Independence Measure (FIM) [37]. The FIM is a rating system of a patient’s ability to perform self-care, sphincter control, mobility, locomotion, communication, social adjustment, and cognition tasks, each of which is rated on a scale between 1 and 7 points depending on the specific degree of assistance required for each task. 

The scale we used for the risk of falls was the Fall Effect Scale [38]. The Falls Scale for the Older Person is an assessment tool designed to identify the older person’s awareness of and practice of behaviors that could potentially protect against falling. People who do not use protective behaviors are potentially at risk of falling, in particular if they are in a group with risk factors for falls such as declining function. The Fall Effect Scale can be self-administered by the older person or administered through an interview, usually taking about 5 to 10 min to complete. It can also be mailed to the person prior to a home visit. Respondents are encouraged to provide a rating (Never, Sometimes, Often, or Always) for each statement and to avoid the “Does not apply” category unless absolutely necessary. This is why we tried to offer “Does not apply” only for those items where it was a possibility. 

The Ashworth scale measures resistance during passive soft-tissue stretching and is used as a simple measure of spasticity [39]. The scale ranges from 0 to 4, where 0 denotes no increase in muscle tone; 1 denotes slight increase in muscle tone, manifested by catch and release or by minimal resistance at the end of the range of motion when the affected part(s) is (are) moved in flexion or extension; 1+ is a slight increase in muscle tone, manifested by a catch, followed by minimal resistance throughout the remainder (less than half) of the Ashworth scale; 2 is a more marked increase in muscle tone through most of the Ashworth scale, but affected part(s) easily move(s); 3 is a considerable increase in muscle tone, but passive movement difficult; and 4 denotes affected part(s) rigid in flexion or extension.

We also used the Dynamic Gait Index (DGI) [40]. DGI is used to assess gait, balance, and fall risk in elderly patients by evaluating usual steady-state walking and walking during more challenging tasks. The intended population is the elderly, stroke patients, and vestibular disorder patients who display poor balance and are at risk of falling. Gait index measures include 8 functional walking tests performed by the patient, and scored on the scale of 0 to 3. Scores of 19 or less are related to increased incidence of falls. The highest score achievable is 24, which indicates safe ambulators. Each question is scored with the lowest category that applies. Time for completion of the exam is 15 min.

As a result of our analysis of the existing assessment scales for the musculoskeletal system functions applied in rehabilitation, a comprehensive scale was developed to assess the nature of the musculoskeletal system damage and the volume of muscle with impaired function (Appendix B). The group with assessed as having disorders of the musculoskeletal system classified as “1” included 6 athletes; “2” included nine athletes; “3” included three athletes; “5” included six athletes; “8” included three athletes; and “9” included six athletes. There were no athletes with estimates of the degree of dysfunction of the musculoskeletal system classified as 5, 7, or 10 points in our study.

Paralympians were tested on the level of psychophysiological functioning. The obtained data were mathematically processed to identify the effect of functional class of athletes and the nature of the musculoskeletal system disorder (the volume of muscle) on psychophysiological functions in two ways: (1) the influence of the functional class of athletes (athletes playing standing; 10 and 6 functional classes were compared) (scale, Appendix A) and (2) the influence of the nature of the musculoskeletal system disorder and the volume of muscle with impaired function (scale, Appendix B).

We evaluated the physical abilities of the Paralympians on this scale with a standard medical examination by athletes before international competitions. We used the data of the 2016 Paralympics. This procedure is standard for all Paralympians.

### 2.4. Methods and Organization of Research

The experiment was conducted in March 2018. To determine the psychophysiological state of athletes during the first and last week of the experiment, psychophysiological indicators were recorded using the computer program Psychodiagnostics (H.S. Skovoroda Kharkiv National Pedagogical University, Kharkiv, Ukraine) [41,42,43]. The following parameters were fixed:

(1) Time a simple visual–motor reaction. Images appear on the monitor screen. The subject should click the left mouse button as soon as he sees the image. Performs 30 attempts. The average value of the reaction time (ms), the standard deviation (ms), the number of errors is recorded.
(2) Choice reaction time (Choice reaction 2–3). Images appear on the monitor screen. The subject must press the left mouse button as soon as he sees the image of the geometric figure. The subject must press the right mouse button as soon as he sees the image of the animal. When other images appear, you do not need to click the mouse button. Performs 30 attempts. The average value of the reaction time (ms), the standard deviation (ms), the number of errors is recorded.
(3) Time complex visual–motor reaction in the feedback mode. The subject must press the left mouse button as soon as he sees the image of the geometric figure. The subject must press the right mouse button as soon as he sees the image of the animal. When other images appear, the subject does not need to click the mouse button. The faster the subject reacts, the faster the next image appears.

The average value of the reaction time (ms), the standard deviation (ms), the number of errors is recorded. In addition, the smallest time the image stays on the screen (the minimum signal exposure time (ms)) is fixed. The time from the start of the test to the subject reaching the peak of the reaction in this test is also recorded (time to reach the minimum signal exposure (s)).

A complex of parameters of a compound visual–motor choice reaction of two of the three elements in feedback mode, that is, as the time reaction changes, the time of signal delivery changes. The “Short version” was used in the feedback mode: the exposure time varies automatically depending on the corresponding reactions of the subject. After the correct answer, the duration of the next signal is reduced by 20 ms, and after the wrong one, the next signal increases by the same amount. The range of the signal exposure change during the test subject’s operation is within 20 to 900 ms with a pause between exposures of 200 ms. The right answer involves pressing the left (right) mouse button while displaying a certain exposure (image), or during a pause after the current exposure. In this test, the time for exciting the minimum exposure of the signal (the time from the start of the test to the subject reaching the peak of the reaction in this test) and the time of the minimum exposure of the signal (the smallest time the image stays on the screen) reflects the functional mobility of the nervous processes (ability to respond quickly to changing situations). The number of errors reflects the strength; the lower the value, the higher the mobility and strength of the nervous system. The duration of the initial exposure is 900 ms, the magnitude of the change in the duration of the signals with correct and therefore erroneous reactions—20 ms (If the subject reacts to the next signal faster than the previous one, the residence time of the next image on the screen is reduced by 20 ms), a pause between the presentation of signals—200 ms, the number of signals—50. The indicators are recorded: the average value of the latent period in ms, deviation in ms, number of errors, run-time test (s), minimum exposure time (ms), and exposure time of minimum exposure (s);

(4) A complex of parameters of a complex visual–motor reaction that involves selecting two of the three elements in feedback mode; as the reaction time changes, the time of signal delivery changes. The “long-term variant” was used in the feedback mode, where the duration of exposure changes automatically depending on the corresponding reactions of the subject. After providing the correct answer, the duration of the next signal is reduced by 20 ms, and after an incorrect response, the duration increases by 20 ms. The range of the signal exposure change during the test subject’s operation is within 20 to 900 ms with a pause between exposures of 200 ms. The correct answer is to press the left (right) mouse button when a certain image is displayed, or during a pause after the current exposure. In this test, the time for achievement the minimum exposure of the signal and the time of the minimum exposure of the signal reflect the functional mobility of the nervous processes. The number of errors reflects the strength nerve processes; the lower the value, the higher the mobility and strength of the nervous system. In addition, the total time of the test reflects a combination of strength and mobility of the nervous system. The duration of the initial exposure is 900 ms, the magnitude of the change in the duration of the signals with correct or erroneous reactions is 20 ms, the pause between the presentations of signals is 200 ms, the number of signals is 120. The indicators are fixed: the average value of the latent period (ms), deviation (ms), number of errors, test runtime (total test time) (s), minimum exposure time (ms), and exit time to minimum exposure (s).

### 2.5. Statistical Analysis 

The computer programs Microsoft Excel-2016 Data Analysis (Version 16, Microsoft, Las Vegas, NV, USA) and SPSS-17 (Version 17.0.3, International Business Machines, Armonk, NY, USA) were applied for statistical processing of the obtained data. We determined the average arithmetic value, the mean square deviation S (SD), and statistical significance according to Student’s *t*-test for each indicator. The dispersion was also analyzed. We determined the influence of the functional class of athletes on the reaction rate in various test modes. We also determined the effect of the upper and lower extremity damage degree on the reaction rate in various test regimes. The degree of influence was considered reliable at a significance level *p* < 0.05. 

Each subject in this file corresponds to a row in the Excel (Microsoft, Las Vegas, USA) Table S1. The columns of the Table S1 show the data of each subject and the results of the tests.

SPSS-17 (Armonk, New York, USA) was used for statistical data processing. Since it is difficult to maintain long names of indicators in the SPSS program, all indicators were abbreviated. Explanation of abbreviations is presented in the Appendix A.

We formulated two assumptions: (1) Psychophysiological indicators significantly differ in athletes of different functional classes, and (2) psychophysiological indicators vary in athletes with different degrees and patterns of lesions of the musculoskeletal system.

To verify these assumptions, we used the following statistical methods:

(1) Analysis of the reliability of differences in the indicators of Paralympians in the functional two functional classes according to Student’s *t*-test (file: Stst.1.sav; file: Interpretation of notation in the program SPSS.docx, Figures S1–S4; file: Stst_T-test_klass.spv).
(2) Analysis of the influence of the Paralympic functional class on psychophysiological indicators (file: Stst.1.sav; file: Interpretation of notation in the program SPSS.docx, file: Stst_Gen_Mod_klass.spv).
(3) Analysis of the reliability of differences in the indicators of groups of athletes with different levels and patterns of lesions of the musculoskeletal system. In this case, more than two independent samples were compared. Therefore, analysis of variance (ANOVA) was used (file: Stst.1.sav; file: Interpretation of notation in the program SPSS.docx, file: Stst_ANOVA_Inc.spv).
(4) Analysis of the impact of the degree and nature of damage to the musculoskeletal system of the Paralympians on psychophysiological indicators. For this, the following actions were performed in SPSS-17 (Armonk, New York, USA) (file: Stst.1.sav; file: Interpretation of notation in the program SPSS.docx, file: Stst_Gen_Mod_inc.spv).

We applied single-factor multivariate dispersion analysis. The indicators of psychophysiological testing were used as dependent variables. The functional class of athletes was applied as the independent variable. To study the influence of the upper or lower extremity damage degree on psychophysiological indicators, we used the point value of the extremity damage degree as an independent variable.

## 3. Results

The performed study confirmed the presence of a significant influence of the athletes’ functional class on the stability of the reaction rate at *p* < 0.05 (indicator “Reaction of choice 2–3”, deviation, ms). In athletes in the 10 functional class, the stability of the reaction is significantly higher in comparison with athletes of the 6 functional class (Table 1 and Table 2). We also detected a significant effect of the functional class on the time to reach the minimum signal exposure (*p* < 0.05) (“Reaction choice in feedback mode, exit time to minimum exposure, s”). In athletes in the 10th functional class, the time for reaching the minimum signal exposure was significant in comparison with athletes in the 6th functional class (Table 1 and Table 2).

We determined the influence of the musculoskeletal system disorder degree on psychophysiological indicators using multidimensional single-factor dispersion analysis. Table 3, Table 4 and Table 5 present of psychophysiological indicators for the athletes in each group according to the developed scale. The obtained data shows that as the dysfunction of the musculoskeletal system increases, the psychophysiological indicators tend to worsen. Some exceptions are the participants with an assessment of dysfunctions of 3 and 8 points (Table 3, Table 4 and Table 5).

The latent period of a simple visual–motor reaction increases with increasing dysfunction of the musculoskeletal system, with the exception of participants with an assessment of dysfunctions of 3 and 8 points (Table 3). The number of errors and the stability of the reaction in the test “simple visual–motor reaction” do not have a pronounced tendency to deteriorate with increasing dysfunction of the musculoskeletal system.

In the test “choice reaction of 2 out of 3 objects”, the psychophysiological indicators tended to deteriorate with increasing degree of dysfunction of the musculoskeletal system (Table 4), with the exception of participants with an assessment of dysfunctions of 8 points, which can be associated with the individual congenital typological features. The choice reaction is key in table tennis [19]; therefore, the identification of a tendency for the results of this test to deteriorate with increasing dysfunction of the musculoskeletal system is an important result for practical work and the implementation of an individual approach for the training of athletes.

We observed the most pronounced tendency deterioration in psychophysiological indicators with increasing dysfunction of the musculoskeletal system in athletes in tests on the reaction of choice in feedback mode (Table 5). This test reflects the mobility of nerve processes [20], which is very important for success in table tennis.

Dispersion analysis with an independent variable “The musculoskeletal system damage degree” showed a significant effect of this indicator on all the studied psychophysiological functions of athletes (Table 3, Table 4, Table 5 and Table 6). As the damage of the locomotor apparatus increases, psychophysiological functions deteriorate at *p* < 0.05, *p* < 0.001 (Table 3, Table 4, Table 5 and Table 6). The lowest rates were observed in athletes with impaired movements of both lower extremities.

The obtained data show that with an increase in the volume of the affected muscles, the psychophysiological functions of athletes decrease. Since motor functions are regulated by the nervous system, an increase in the volume of affected muscles should influence the mobility of the nervous processes. Many diseases associated with disorders of the musculoskeletal system also include disorders of the nervous system. Therefore, in the training process, it is important to consider not only their functional class, but also the nature of the musculoskeletal system damage, the volume of the affected muscles, and the fact that damage to the lower extremities affects psychophysiological indicators more than the damage to the upper extremities or unilateral damage.

## 4. Discussion

In this study, we confirmed our hypothesis concerning the influence of musculoskeletal system disorder degree on the psychophysiological functioning of Paralympians in terms of the characteristics and degree of motor disorder of the upper and lower extremities. The hypothesis was partially confirmed regarding the influence of belonging to a certain functional class of Paralympians in table tennis. The first assumption that psychophysiological indicators significantly differ in athletes of different functional classes was partially confirmed. The second assumption of psychophysiological indicators differing in athletes with different degrees and nature of the musculoskeletal system was fully confirmed.

The obtained data allow us to conclude that elite athletes, Paralympians specializing in table tennis, do not differ according to their functional class in most psychophysiological indicators. The significant impact of the functional class was revealed only by two indicators: “Choice reaction 2–3, deviation (ms)” and “Choice reaction in feedback mode, time to minimum signal exposure, s”. In the test use to determine the choice of 2 out of 3 objects, it is necessary to press the left mouse button when the geometric shape appeared on the screen and to press the right mouse button when the image from the animal world. We determined the average test run time for each test participant based on 50 attempts. We also determined the number of errors and the standard deviation for 50 attempts for each test participant. A significant impact of the functional class of athletes was found for indicators “Choice reaction 2–3, deviation (ms)” and “Choice reaction in feedback mode, time to minimum signal exposure, s”. Table 1 shows that the standard deviation was smaller for athletes in the 10th class. This means that the stability of the latent time of a compound reaction depends on the functional class of Paralympians athletes in table tennis.

Another indicator that shows the influence of athletes’ functional class on the test result is “Choice reaction in the feedback mode, time to reach the minimum signal exposure, s”. This test was performed in feedback mode: the faster the subject reacts to the signal, the faster the next signal appears. The faster the participant reaches the individual maximum when performing this test (i.e., the faster they reach smallest signal exposure time), the higher the mobility of the nervous processes. The obtained data confirms that athletes with minimal musculoskeletal system damage have a maximum reaction rate faster than athletes with more serious disorders. We proved that the musculoskeletal system also affects the mobility of the nervous processes because the nervous system regulates muscle activity. Therefore, muscular system disorders change the working of the nervous system. In Paralympic athletes, some disorders initially affect both the muscular system and the nervous system (e.g., cerebral palsy). Therefore, identifying the effect of the musculoskeletal system disorders on the working of the nervous system is important.

However, according to the obtained data, the other studied parameters are not influenced by the athletes’ functional class (Table 2). This may be due to the fact that athletes who are similar in their ability to hold a tennis racket and stand near the tennis table are combined in one functional class. However, these athletes may differ in the nature of the musculoskeletal system disorder and the volume of the affected muscle. These factors are related to the nervous system activity and should affect the psychophysiological indicators. Therefore, we analyzed the influence of the degree and nature of the musculoskeletal system disorder on the psychophysiological functions of Paralympians. The musculoskeletal system disorder degree, in this case, was determined by a specially developed scale based on the existing scales in rehabilitation: muscular strength [35], balance assessment [36], functional capacity determination [37], risk of falls [38], and other [39]. In our opinion, these scales provide a more differentiated assessment of the musculoskeletal system functioning in comparison with the functional classification of Paralympian athletes in table tennis, since the functional classification is based mainly on the principle of considering the ability to play table tennis without a detailed consideration of the nature of the impairment [30].

Therefore, we further aimed to identify the influence of the nature of the musculoskeletal system disorder on a special scale that we developed on the basis of the scales existing in rehabilitation. 

We revealed that belonging to a certain functional class of Paralympians only affects the stability of the reaction rate and the achievement time for the minimum signal exposure in the test for the choice reaction rate in feedback mode at *p* < 0.05. In this test, the signals are delivered the faster, the shorter is the reaction time of the participant to the signal. The faster the subject reacts, the faster the next image appears. The faster an athlete reaches their minimum signal exposure time, the higher the mobility of their nervous system. This means that in the central nervous system, the switching of the work from some nerve centers to others is faster. Dispersion analysis showed that athletes in the 10 functional class respond faster compared to athletes in the 6 functional class. In addition, athletes of the 10 functional class have a higher stability in the reaction rate to visual stimuli. However, no significant effect was revealed between athlete’s functional class and reaction time, number of errors, or stability in a simple reaction to a visual signal. In addition, there was no significant effect of the athletes’ functional class on the reaction time or in the number of errors in the choice reaction of two objects out of three. The same was true for the test of the choice reaction in feedback mode: the reaction time, the number of errors, the stability of the reactions, and the minimum signal exposure time do not depend on the functional class of athletes. The obtained data are partially consistent with studies by Van Biesenet et al. [20] and Santos et al. [24]. Our studies showed that only a small part of the psychophysiological functioning depends on the functional class of table tennis Paralympians. However, when expressing functional disorders irrespective of the functional class of Paralympians, but on conditional scores of damage of the upper and lower extremity, a significant effect of the disorder degree on all studied psychophysiological indicators was revealed with *p* < 0.001, *p* < 0.05 with respect to damage of the upper and lower extremity. This means that the speed of reaction to a visual signal, the number of errors during the test of reaction speed, and the mobility of nervous processes in table tennis Paralympians depend on the upper and lower extremity damage degree, but practically does not depend on the functional class of Paralympians in table tennis. The worst psychophysiological results were observed in athletes with disabilities in both lower extremities. Unilateral damage to the extremities and congenital underdevelopment of the extremities had a lesser effect on the psychophysiological functions [44,45,46].

The obtained data are new in the study of psychophysiological functioning of Paralympic athletes. We revealed that a higher damage degree of the upper and lower extremities influences the psychophysiological functions of Paralympic table tennis athletes in comparison with the influence of functional classification. Our results support the need to consider the characteristics of the upper and lower extremity damage in the functional classification of Paralympians. In addition, the functional classification of Paralympic table tennis athletes should consider the level of psychophysiological functioning.

These provisions are important for the competitions in the Paralympian sport, in particular, table tennis. The obtained data are important for structuring the training process of Paralympians. The results that show the influence of the upper and lower extremities damage degree on the psychophysiological functioning indicate the need for an individual approach to table tennis athlete training. The results show that when training Paralympics athletes in table tennis, it is important to consider not only their functional class but also the upper and lower extremities damage degree and the level of psychophysiological functioning. Our data complete the concept of an individual approach to sports with these provisions [47,48,49].

The obtained data contribute to the study of the relationship between motor and psychological functioning, showing that disorders of the motor apparatus are interrelated with the deterioration of the nervous system. The malfunction of the lower extremities has a more pronounced effect on the working of the nervous system compared with disorders of the upper extremities and unilateral damage to the musculoskeletal system.

The results also confirm the integrity of the body functioning and the connection between consciousness and motor actions [50]. Restriction of motor actions affects the functioning of the nervous system and consciousness. In turn, disorders of the nervous system in the form of cerebral palsy affect psychophysiological functioning (reaction rate and the mobility of the nervous system) and the locomotor apparatus. Damage of the lower extremities is associated with a more expressed decrease in psychophysiological functioning compared with upper extremities disorders, unilateral damage to the extremities, and congenital anomaly of the extremities.

## 5. Conclusions 

Belonging to a certain functional class of athlete’s influences the stability of the reaction rate and the time to reach the minimum signal exposure in the speed test for a choice reaction with feedback. Athletes of in the 10 functional class are reliably faster in the minimum signal exposure test compared with athletes in the 6 functional class.

There was no significant effect of athlete functional class on the reaction time, the number of errors, and stability in a simple reaction to a visual signal. In addition, there was no significant effect of functional class on the reaction time or the number of errors in the choice reaction of two objects out of three. The same is true for the test of the choice reaction with feedback: the reaction time, the number of errors, the stability of the reactions, and the signal exposure time do not depend reliably on the functional class of athletes.

The speed of reaction to a visual signal, the number of errors during the test for reaction speed, and the mobility of nervous processes in Paralympian table tennis athletes depend on the degree of damage to the upper and lower extremities. The worst results in psychophysiological indicators were found in athletes with disabilities in both lower extremities. Unilateral damage to the extremities and congenital underdevelopment of the extremities had less effect on the psychophysiological functions.

The revealed higher degree of influence of the upper and lower extremities degree of damage on the psychophysiological functions of Paralympians in table tennis in comparison with the influence of the functional class indicates the need to consider the functional classification of these athletes including features of the damage to the upper and lower extremities. In addition, in the functional classification of Paralympian table tennis athletes should consider the level of psychophysiological functioning of athletes.

When training Paralympic athletes in table tennis, it is important to consider their functional class as well as the upper and lower extremities degree of damage and the level of psychophysiological functioning.

## 6. Limitations

The results of this study apply only to athletes who specialize in table tennis. The subjects in this study were Paralympians who compete in international competitions: the World Cup, the Paralympic Games. The results of this study do not apply to beginner athletes with disorders of musculoskeletal system, as well as to athletes without disorders of musculoskeletal system. The results of this study do not apply to Paralympic other sports. The study of the characteristics of the reaction rate in various modes of testing athletes with disorders of musculoskeletal system in other sports requires additional research.

## Figures and Tables

**Table 1:** Psychophysiological indicators of elite table tennis athletes with the musculoskeletal system disorders of various functional classes.

Indicator	Group Statistics					
	Class	Mean	SD	SEM	t	Sig. (2-tailed)
Simple visual–motor reaction, time of the latent period (ms)	10	357.83	37.80	8.91	–1.252	0.22
	6	375.00	40.89	10.56	–	–
Simple visual–motor reaction errors (number)	10	0.33	0.77	0.18	–1.152	0.258
	6	0.60	0.51	0.13	–	–
Simple visual–motor reaction, deviation (ms)	10	2.78	0.04	0.01	–0.938	0.356
	6	2.79	0.03	0.01	–	–
Choice reaction 2–3, time of latent period (ms)	10	596.83	50.12	11.81	–1.339	0.19
	6	623.80	65.55	16.92	–	–
Choice reaction 2–3, errors (number)	10	16.00	3.66	0.86	–1.095	0.282
	6	18.00	6.64	1.72	–	–
Choice reaction 2–3, deviation (ms)	10	4.26	0.64	0.15	–2.172	0.038
	6	5.01	1.30	0.34	–	–
Choice reaction in feedback mode, length of latent period (ms)	10	515.50	36.28	8.55	0.007	0.994
	6	515.40	45.63	11.78	–	–
Choice reaction in feedback mode, errors (number)	10	34.83	6.27	1.48	0.653	0.519
	6	32.80	11.31	2.92	–	–
Choice reaction in feedback mode, deviation (ms)	10	4.56	0.54	0.13	1.136	0.265
	6	4.35	0.52	0.13	–	–
Choice reaction in feedback mode, minimum exposure time (ms)	10	780.00	158.97	37.47	1.072	0.292
	6	716.00	183.96	47.50	–	–
Choice reaction in feedback mode, total test time (s)	10	158.33	34.62	8.16	0.59	0.56
	6	150.80	38.77	10.01	–	–
Choice reaction in feedback mode, achievement time to minimum exposure (s)	10	41.5	21.89	5.1616	–2.33	0.027
	6	56.2	11.79	3.04443	–	–

Note: Mean—average value; t—Student’s *t*-test criterion; Sig—significance.

**Table 2:** The results of dispersion analysis of the influence of functional class on the psychophysiological indicators of elite table tennis athletes with musculoskeletal system disorders.

Source	Dependent Variable	Tests of Between-Subjects Effects							
		Type III Sum of Squares	*R* ^2^	Adjusted *R*^2^	df	Mean Square	F	Sig.	Partial Eta^2^
**Corrected Model**	Simple visual–motor reaction, time of the latent period (ms)	2411.136	0.048	0.017	1	2411.14	1.57	0.22	0.048
	Simple visual–motor reaction errors (number)	0.582	0.041	0.010	1	0.58	1.33	0.258	0.041
	Simple visual–motor reaction, deviation (ms)	0.001	0.037	0.006	1	0.00	1.19	0.284	0.037
	Choice reaction 2–3, time of latent period (ms)	5949.827	0.055	0.024	1	5949.83	1.79	0.19	0.055
	Choice reaction 2–3, errors (number)	32.727	0.037	0.006	1	32.73	1.20	0.282	0.037
	Choice reaction 2–3, deviation (ms)	4.621	0.132	0.104	1	4.62	4.71	0.038	0.132
	Choice reaction in feedback mode, length of latent period (ms)	0.082	0.000	0.032	1	0.08	0.00	0.994	0.00
	Choice reaction in feedback mode, errors (number)	33.827	0.014	0.018	1	33.83	0.43	0.519	0.014
	Choice reaction in feedback mode, deviation (ms)	0.356	0.040	0.009	1	0.36	1.29	0.265	0.04
	Choice reaction in feedback mode, minimum exposure time (ms)	33,512.727	0.036	0.005	1	33,512.73	1.15	0.292	0.036
	Choice reaction in feedback mode, total test time (s)	464.327	0.011	0.021	1	464.33	0.35	0.56	0.011
	Choice reaction in feedback mode, exit time to minimum exposure (s)	1768.009	0.149	0.122	1	1768.01	5.43	0.027	0.149
**Functional class**	Simple visual–motor reaction, time of the latent period (ms)	2411.136			1	2411.14	1.57	0.220	0.048
	Simple visual–motor reaction errors (number)	0.582			1	0.58	1.33	0.258	0.041
	Simple visual–motor reaction, deviation (ms)	0.001			1	0.00	1.19	0.284	0.037
	Choice reaction 2–3”, time of latent period (ms)	5949.827			1	5949.83	1.79	0.19	0.055
	Choice reaction 2–3, errors (number)	32.727			1	32.73	1.20	0.282	0.037
	Choice reaction 2–3, deviation (ms)	4.621			1	4.62	4.71	0.048	0.132
	Choice reaction in feedback mode, length of latent period (ms)	0.082			1	0.08	0.00	0.994	0
	Choice reaction in feedback mode, errors (number)	33.827			1	33.83	0.43	0.519	0.014
	Choice reaction in feedback mode, deviation (ms)	0.356			1	0.36	1.29	0.265	0.04
	Choice reaction in feedback mode, minimum exposure time (ms)	33,512.727			1	33,512.73	1.15	0.292	0.036
	Choice reaction in feedback mode, total test time (s)	464.327			1	464.33	0.35	0.56	0.011
	Choice reaction in feedback mode, exit time to minimum exposure (s)	1768.009			1	1768.01	5.43	0.027	0.149

Note: F—Fisher criterion; Sig—significance; R—The square of R is the value of the dependent variable of the variance, which is taken into account by the adjusted model; df—degrees of freedom; Eta^2^—correlation ratio. It is used to express the degree of influence or the strength of the effect X (independent variable, factor) on Y (dependent variable). The value of Eta-square lies in the range from 0 to 1.

**Table 3:** Psychophysiological indicators of elite athletes in table tennis with musculoskeletal system disorders with different degrees of the upper and lower extremity damage (“Simple visual–motor reaction”).

Name of Metrics	Degree of Musculoskeletal Dysfunction	Descriptive Statistics		ANOVA					
		Mean	SD		Sum of Squares	df	Mean Square	F	Sig.
Simple visual–motor reaction, time of the latent period (ms)	1	352.50	31.22	Between Groups	40,306.64	5	8061.327	22.194	0.000
	2	383.00	10.82	Within Groups	9807	27	363.222	–	–
	3	293.00	0.00	Total	50,113.64	32	–	–	–
	5	374.00	26.29	–	–	–	–	–	–
	8	310.00	0.00	–	–	–	–	–	–
	9	408.50	10.41	–	–	–	–	–	–
	Total	365.64	39.57	–	–	–	–	–	–
Simple visual–motor reaction errors (number)	1	0.00	0.00	Between Groups	12.682	5	2.536	45.655	0.000
	2	0.00	0.00	Within Groups	1.5	27	0.056	–	–
	3	2.00	0.00	Total	14.182	32	–	–	–
	5	1.00	0.00	–	–	–	–	–	–
	8	0.00	0.00	–	–	–	–	–	–
	9	0.50	0.55	–	–	–	–	–	–
	Total	0.45	0.67	–	–	–	–	–	–
Simple visual–motor reaction, deviation (ms)	1	2.76	0.00	Between Groups	0.032	5	0.006	47.528	0.000
	2	2.76	0.00	Within Groups	0.004	27	0	–	–
	3	2.87	0.00	Total	0.035	32	–	–	–
	5	2.81	0.00	–	–	–	–	–	–
	8	2.76	0.00	–	–	–	–	–	–
	9	2.79	0.03	–	–	–	–	–	–
	Total	2.79	0.03	–	–	–	–	–	–

**Table 4:** Psychophysiological indicators of elite table tennis athletes with musculoskeletal system disorders (“choice reaction 2 of 3”).

Metrics	Degree of Musculoskeletal Dysfunction	Descriptive Statistics		ANOVA					
		Mean	SD		Sum of Squares	df	Mean Square	F	Sig.
Choice reaction 2–3, time of latent period (ms)	1	544.00	18.62	Between Groups	57,311.23	5	11,462.25	6.011	0.001
	2	570.33	50.80	Within Groups	51,489.5	27	1907.019	–	–
	3	582.00	0.00	Total	108,800.7	32	–	–	–
	5	666.50	30.12	–	–	–	–	–	–
	8	542.00	0.00	–	–	–	–	–	–
	9	622.00	70.11	–	–	–	–	–	–
	Total	609.09	58.31	–	–	–	–	–	–
Choice reaction 2–3, errors (number)	1	12.50	1.64	Between Groups	773.227	5	154.645	39.578	0.000
	2	12.67	1.32	Within Groups	105.5	27	3.907	–	–
	3	19.00	0.00	Total	878.727	32	–	–	–
	5	21.00	3.29	–	–	–	–	–	–
	8	6.00	0.00	–	–	–	–	–	–
	9	21.00	2.19	–	–	–	–	–	–
	Total	16.91	5.24	–	–	–	–	–	–
Choice reaction 2–3, deviation (ms)	1	4.88	0.42	Between Groups	24.09	5	4.818	11.88	0.000
	2	3.69	0.15	Within Groups	10.947	27	0.405	–	–
	3	4.71	0.00	Total	35.037	32	–	–	–
	5	5.57	1.19	–	–	–	–	–	–
	8	3.10	0.00	–	–	–	–	–	–
	9	5.39	0.75	–	–	–	–	–	–
	Total	4.60	1.05	–	–	–	–	–	–

**Table 5:** Psychophysiological indicators of elite table tennis athletes with disorders of the musculoskeletal system with varying degrees of lesions of the upper and lower extremities (The reaction time of the choice in the feedback mode).

Name of Metrics	Degree of Musculoskeletal Dysfunction	Descriptive Statistics		ANOVA					
		Mean	SD		Sum of Squares	df	Mean Square	F	Sig.
Choice reaction in feedback mode, length of latent period (ms)	1	464.00	14.24	Between Groups	33,130.68	5	6626.136	9.727	0.000
	2	491.67	4.77	Within Groups	18,393.5	27	681.241	–	–
	3	490.00	0.00	Total	51,524.18	32	–	–	–
	5	539.00	48.20	–	–	–	–	–	–
	8	462.00	0.00	–	–	–	–	–	–
	9	518.50	33.41	–	–	–	–	–	–
	Total	515.45	40.13	–	–	–	–	–	–
Choice reaction in feedback mode, errors (number)	1	31.50	2.74	Between Groups	2001.727	5	400.345	22.015	0.000
	2	31.67	7.47	Within Groups	491	27	18.185	–	–
	3	37.00	0.00	Total	2492.727	32	–	–	–
	5	25.50	0.55	–	–	–	–	–	–
	8	21.00	0.00	–	–	–	–	–	–
	9	46.00	1.10	–	–	–	–	–	–
	Total	33.91	8.83	–	–	–	–	–	–
Choice reaction in feedback mode, deviation (ms)	1	4.78	0.72	Between Groups	4.286	5	0.857	4.972	0.002
	2	4.42	0.48	Within Groups	4.655	27	0.172	–	–
	3	4.54	0.00	Total	8.94	32	–	–	–
	5	3.97	0.13	–	–	–	–	–	–
	8	3.94	0.00	–	–	–	–	–	–
	9	4.94	0.19	–	–	–	–	–	–
	Total	4.46	0.53	–	–	–	–	–	–
Choice reaction in feedback mode, minimum exposure time (ms)	1	790.00	10.95	Between Groups	587672.7	5	117534.5	9.088	0.000
	2	720.00	199.75	Within Groups	349200	27	12933.33	–	–
	3	740.00	0.00	Total	936872.7	32	–	–	–
	5	670.00	76.68	–	–	–	–	–	–
	8	440.00	0.00	–	–	–	–	–	–
	9	900.00	0.00	–	–	–	–	–	–
	Total	750.91	171.11	–	–	–	–	–	–
Choice reaction in feedback mode, total test time (s)	1	186.00	14.24	Between Groups	28355.73	5	5671.145	11.32	0.000
	2	144.00	39.47	Within Groups	13527	27	501	–	–
	3	146.00	0.00	Total	41882.73	32	–	–	–
	5	128.50	2.74	–	–	–	–	–	–
	8	106.00	0.00	–	–	–	–	–	–
	9	195.50	1.64	–	–	–	–	–	–
	Total	154.91	36.18	–	–	–	–	–	–
Choice reaction in feedback mode, exit time to minimum exposure (s)	1	35.00	24.10	Between Groups	3608.909	5	721.782	2.36	0.067
	2	49.67	22.36	Within Groups	8258	27	305.852	–	–
	3	30.00	0.00	Total	11866.91	32	–	–	–
	5	49.00	16.43	–	–	–	–	–	–
	8	57.00	0.00	–	–	–	–	–	–
	9	63.00	1.10	–	–	–	–	–	–
	Total	48.18	19.26	–	–	–	–	–	–

**Table 6:** The results of dispersion analysis of the influence of the upper and lower extremity damage degree on the psychophysiological indicators of table tennis Paralympians (fragment, full version in Annex 3).

Source	Dependent Variable	Tests of Between-Subjects Effects							
		Type III Sum of Squares	*R* ^2^	Adjusted *R*^2^	df	Mean Square	F	Sig.	Partial Eta Squared
Corrected Model	Simple visual–motor reaction, time of the latent period (ms)	40306.636	0.017	0.768	5	8061.33	22.19	0.00	0.804
	Simple visual–motor reaction errors (number)	12.682	0.010	0.875	5	2.54	45.66	0.00	0.894
	Simple visual–motor reaction, deviation (ms)	0.032	0.006	0.879	5	0.01	47.53	0.00	0.898
	Choice reaction 2–3”, time of latent period (ms)	57,311.227	0.024	0.439	5	11,462.25	6.01	0.00	0.527
	Choice reaction 2–3, errors (number)	773.227	0.006	0.858	5	154.65	39.58	0.00	0.88
	Choice reaction 2–3, deviation (ms)	24.090	0.104	0.630	5	4.82	11.88	0.00	0.688
	Choice reaction in feedback mode, length of latent period (ms)	33,130.682	0.032	0.577	5	6626.14	9.73	0.00	0.643
	Choice reaction in feedback mode, errors (number)	2001.727	0.018	0.767	5	400.35	22.02	0.00	0.803
	Choice reaction in feedback mode, deviation (ms)	4.286	0.009	0.383	5	0.86	4.97	0.00	0.479
	Choice reaction in feedback mode, minimum exposure time (ms)	587672.727	0.005	0.558	5	117,534.55	9.09	0.00	0.627
	Choice reaction in feedback mode, total test time (s)	28,355.727	0.021	0.617	5	5671.15	11.32	0.00	0.677
	Choice reaction in feedback mode, exit time to minimum exposure (s)	3608.909	0.122	0.175	5	721.78	2.56	0.04	0.304
Degree of musculoskeletal dysfunction	Simple visual–motor reaction, time of the latent period (ms)	40,306.636	–	–	5	8061.33	22.19	0.00	0.804
	Simple visual–motor reaction errors (number)	12.682	–	–	5	2.54	45.66	0.00	0.894
	Simple visual–motor reaction, deviation (ms)	0.032	–	–	5	0.01	47.53	0.00	0.898
	Choice reaction 2–3”, time of latent period (ms)	57,311.227	–	–	5	11,462.25	6.01	0.00	0.527
	Choice reaction 2–3, errors (number)	773.227	–	–	5	154.65	39.58	0.00	0.88
	Choice reaction 2–3, deviation (ms)	24.09	–	–	5	4.82	11.88	0.00	0.688
	Choice reaction in feedback mode, Time of latent period (ms)	33,130.682	–	–	5	6626.14	9.73	0.00	0.643
	Choice reaction in feedback mode, errors (number)	2001.727	–	–	5	400.35	22.02	0.00	0.803
	Choice reaction in feedback mode, deviation (ms)	4.286	–	–	5	0.86	4.97	0.00	0.479
	Choice reaction in feedback mode, minimum exposure time (ms)	587,672.72	–	–	5	117,534.55	9.09	0.00	0.627
	Choice reaction in feedback mode, total test time (s)	28,355.727	–	–	5	5671.15	11.32	0.00	0.677
	Choice reaction in feedback mode, achievement time to minimum exposure (s)	3608.909	–	–	5	721.78	2.56	0.04	0.304

## Supplementary Materials

The following are available online at https://www.mdpi.com/2075-4663/7/3/55/s1, Table S1: Diagnost.xlsx; Stst.1.sav; Stst_ANOVA_Inc.spv; Stst_Gen_Mod_inc.spv; Stst_Gen_Mod_klass.spv; Stst_T-test_klass.spv; Figures: in file: Interpretation of notation in the program SPSS.docx; Video: Psyhodiagnost.avi; Video Abstract 1.mp4.

## Appendix A

**Table A1:** Minimum damage for athletes who compete in a standing position with cerebral palsy, amputations, and other lesions of the musculoskeletal system [30].

Class	Damage
Class 10 (minimal disabilities standing classes)	● single stiff ankle
	● amputation of forefoot through all metatarsals (minimal 1/3 of foot amputated)
	● hip (sub)luxation
	● moderate to mild reduction of ROM in the major joints
	● polio: loss of 10 points in muscles strength in one lower extremity distributed over the whole leg
	● polio: 10 points of loss over two legs is not considered to meet the minimal disability or Very mild impairment of playing arm
	● finger amputation/dysmelia with functional grip (more than 4 phalanges loss—thumb not taken in consideration)
	● stiff wrist with functional grip
	● weakness of the hand or a joint of the arm ITTF PARA TABLE TENNIS DIVISION A Committee of the International Table Tennis Federation 31 or Severe to moderate impairment of non-playing arm
	● single BE with a stump length not longer than 2/3 of forearm (the forearm = the length of the ulna)
	● brachial plexus lesion with some residual functions
	● dysmelia or similar disabilities not longer than 2/3 of the forearm or Moderate impairment of the trunk ● stiffness (ankylosing spondylitis)
	● extreme curvatures of the back (kyphosis, scoliosis, kyphoscoliosis, hyperlordosis) ● fusion ● muscular dystonia with effects on the spine or Any disability with comparable functional profile Nanism is recognized as a disability
	● athletes with Nanism start in class 10 but as a result of other impairments, they may be considred for a lower class, e.g., normally a player with a single BK amputation is class 9 but Nanism plus BK amputation is class 8
	● body length: male: 140 cm and less female: 137 cm and less
Class 6: Severe impairments of legs and arms	● severe Cerebral Palsy (CP)—hemiplegia with playing arm included
	● severe CP—diplegia playing arm included
	● severe CP—athetoid (involuntary slow movements)—abnormal strokes—poor balance—poor movements
	● amputation on playing arm and leg(s) or both arms and leg(s) or similar dysmelia ● double above knee amputation (double AK)
	● arthrogryposis playing arm and leg(s) or both arms and leg(s)
	● muscular dystrophy of limbs and trunk or other neuromuscular disability of comparable impairment profile
	● incomplete spinal cord injury of comparable profile
	● a player with the handle of the racket in his or her mouth
	● any disability with comparable functional profile

## Appendix B

**Table A2:** Developed scale for assessing the musculoskeletal system damage according to the nature of the disease and the volume of the affected muscles.

Group	Impairment
1	one hand damage
2	spinal curvature
3	two hands damage
4	muscular dystonia
5	hemiplegia, hand and legs functions partially preserved
6	hemiplegia, leg functions partially preserved, hand functions significantly impaired
7	muscular dystrophy, hands and legs motions and partially saved
8	congenital anomalies of the upper and lower extremities
9	diplegia with a strong violation of legs motions
10	the athlete plays with his mouth
