# Supplementary material for: Influence of Musculoskeletal System Dysfunction Degree on Psychophysiological Indicators of Paralympic Athletes
Source: Sports (Basel). 2019 Feb 26;7(3):55. doi: 10.3390/sports7030055 (PMC6473616; doi:10.3390/sports7030055)
Supplement: Supplementary file 1 [file sports-07-00055-s001.zip › sports-403903-SI/Interpretation of notation in the program SPSS.docx]

**Table S1.** Interpretation of notation in the program SPSS.

| **Notation** | **Interpretation** |
| --- | --- |
| класс | functional classes |
| ПЗМР_врем | "Simple visual-motor reaction", time of the latent period, ms |
| ПЗМР_ош | "Simple visual-motor reaction" errors, number |
| ПЗМР_откл | "Simple visual-motor reaction", deviation, ms |
| РВ2_3_врем | “Choice reaction 2-3”, time of latent period, ms |
| РВ2_3_ош | " Choice reaction 2-3", errors, number |
| РВ2_3_откл | " Choice reaction 2-3", deviation, ms |
| УФПНС_врем | "Choice reaction in the feedback mode", Time of the latent period, ms |
| УФПНС_ош_ | "Choice reaction in the feedback mode", errors, number |
| УФПНС_от_ | " Choice reaction in the feedback mode", deviation, ms |
| УФПНС_мин.вр.эксп | "Choice reaction in the feedback mode", minimum exposure time, ms |
| УФПНС_общ.время_вып | "Choice reaction in the feedback mode", the total test time, ms |
| УФПНС_вр_вых_МЭ | "Choice reaction in the feedback mode", exit time to minimum exposure, s |
| тяжесть_бол | Degree of musculoskeletal dysfunction |


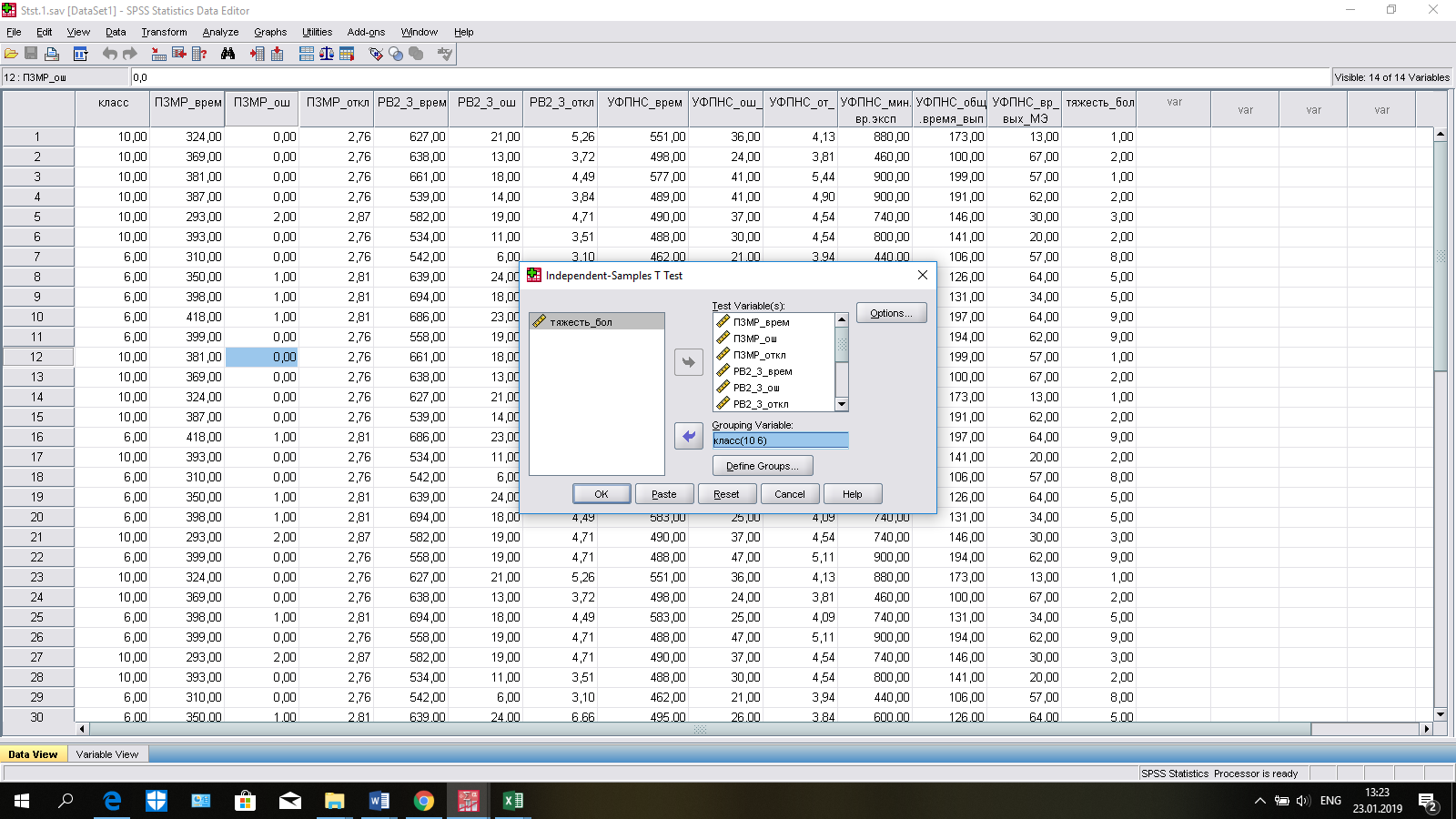


**Figure S1.**


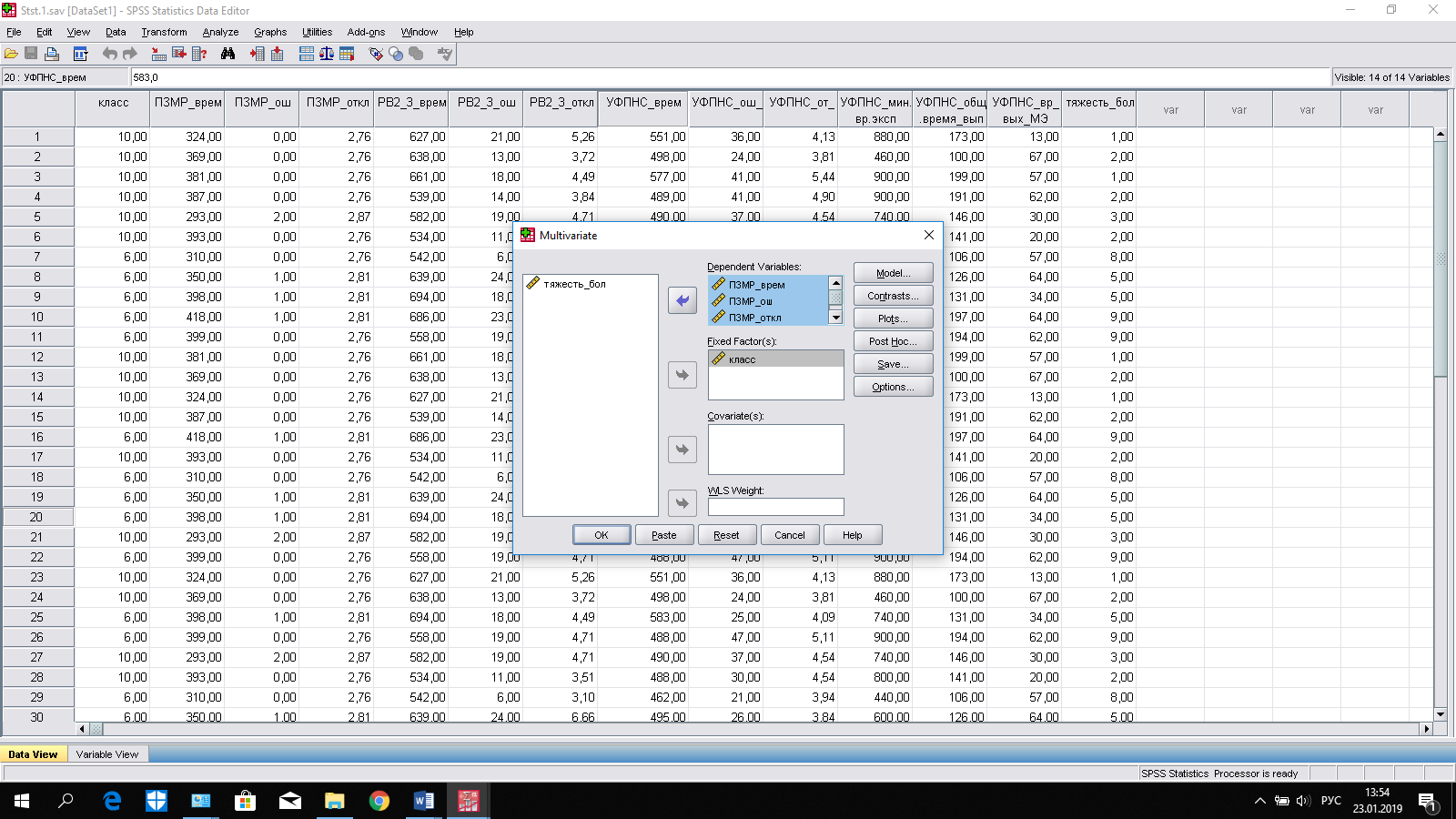


**Figure S2.**


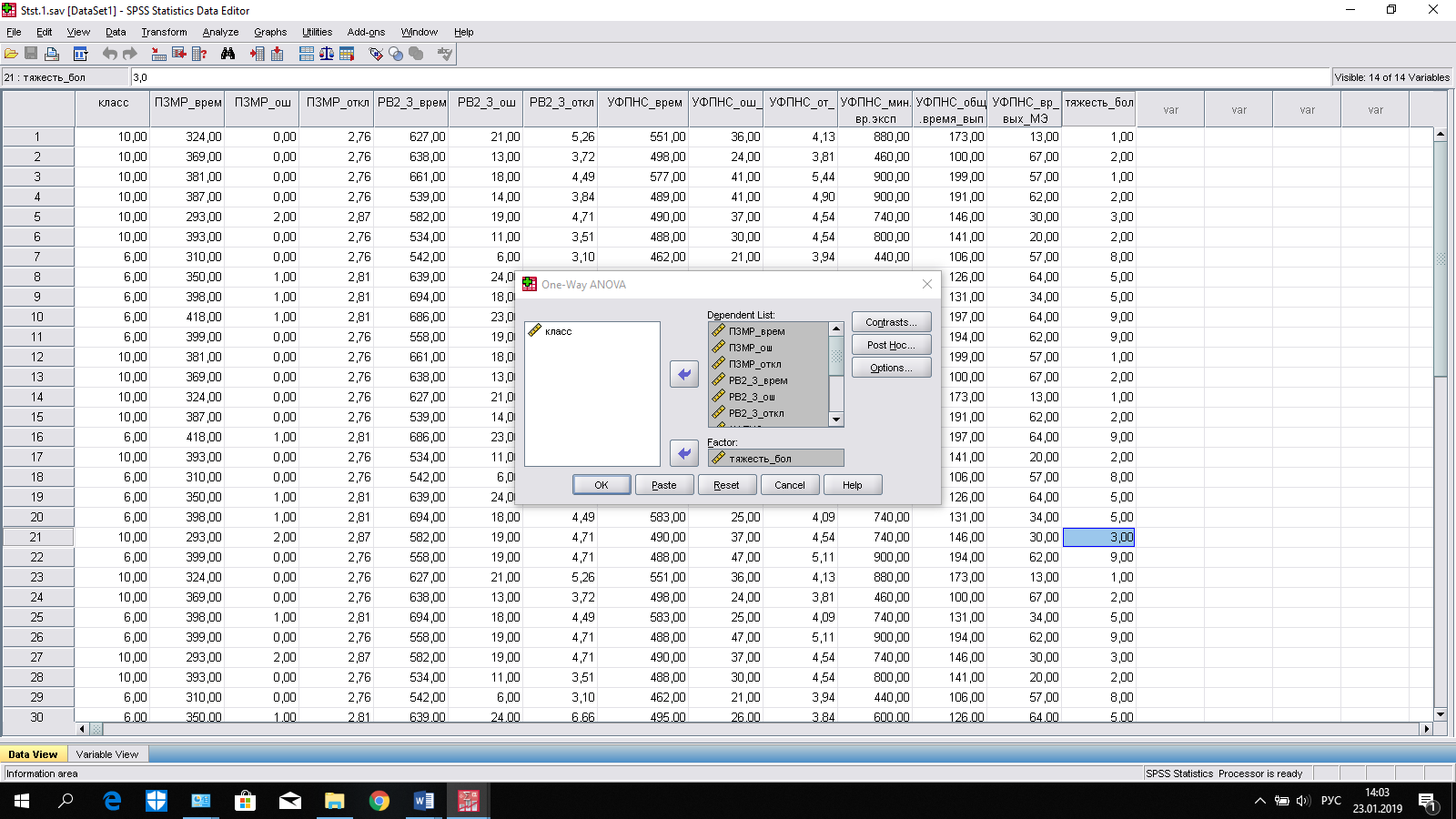


**Figure S3.**


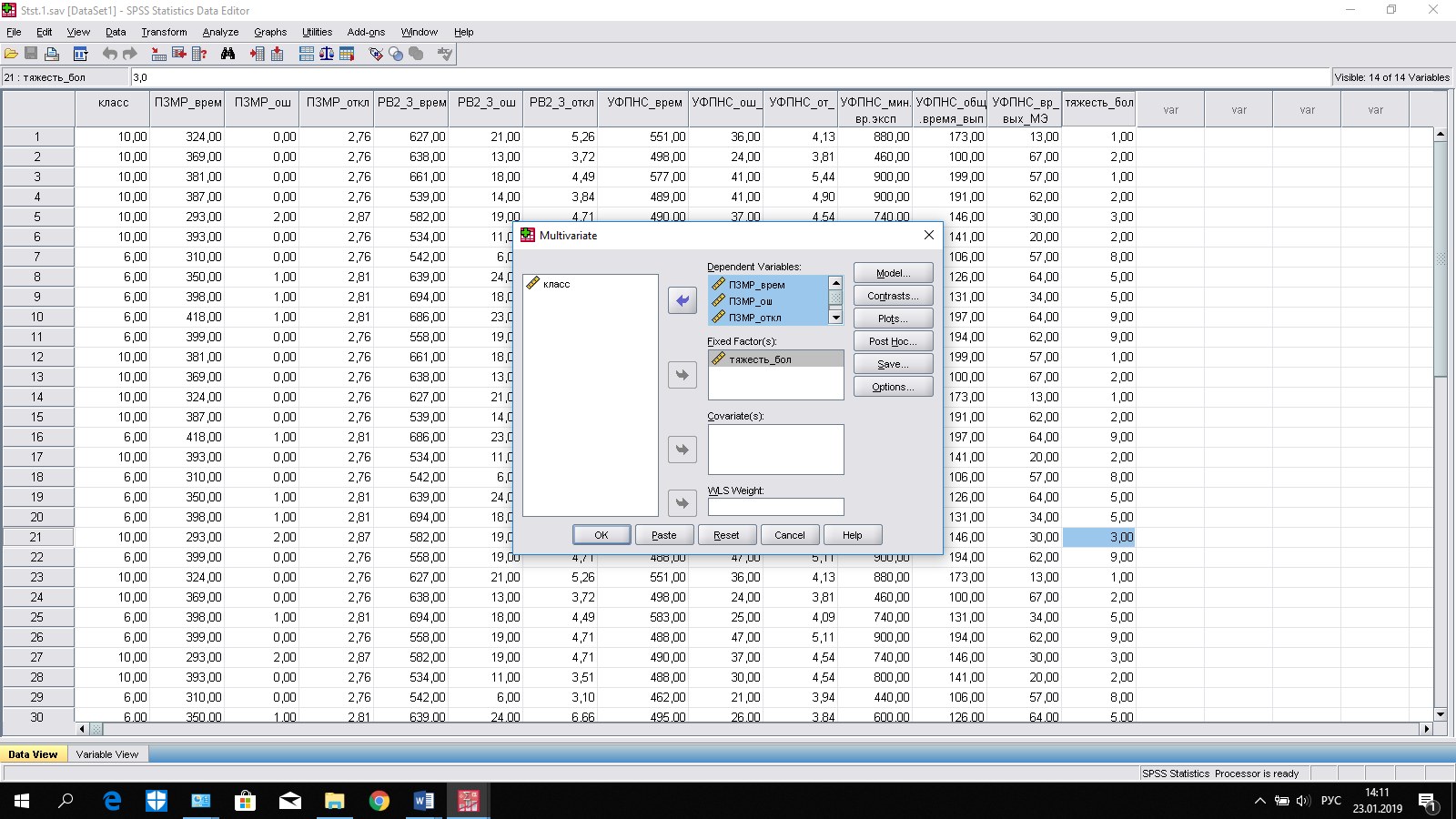


**Figure S4.**
